# Cannabinoids induce functional Tregs by promoting tolerogenic DCs via autophagy and metabolic reprograming

**DOI:** 10.1038/s41385-021-00455-x

**Published:** 2021-09-21

**Authors:** Alba Angelina, Mario Pérez-Diego, Jacobo López-Abente, Beate Rückert, Ivan Nombela, Mübeccel Akdis, Mar Martín-Fontecha, Cezmi Akdis, Oscar Palomares

**Affiliations:** 1 Department of Biochemistry and Molecular Biology, School of Chemistry, Complutense University of Madrid, Madrid, Spain; 2 Swiss Institute of Allergy and Asthma Research (SIAF), University of Zürich, Davos, Switzerland; 3 Department of Organic Chemistry, School of Optics and Optometry, Complutense University of Madrid, Madrid, Spain

## Abstract

The generation of functional regulatory T cells (Tregs) is essential to keep tissue homeostasis and restore healthy immune responses in many biological and inflammatory contexts. Cannabinoids have been pointed out as potential therapeutic tools for several diseases. Dendritic cells (DCs) express the endocannabinoid system, including the cannabinoid receptors CB1 and CB2. However, how cannabinoids might regulate functional properties of DCs is not completely understood. We uncover that the triggering of cannabinoid receptors promote human tolerogenic DCs that are able to prime functional FOXP3^+^ Tregs in the context of different inflammatory diseases. Mechanistically, cannabinoids imprint tolerogenicity in human DCs by inhibiting NF-κB, MAPK and mTOR signalling pathways while inducing AMPK and functional autophagy flux via CB1- and PPARα-mediated activation, which drives metabolic rewiring towards increased mitochondrial activity and oxidative phosphorylation. Cannabinoids exhibit in vivo protective and anti-inflammatory effects in LPS-induced sepsis and also promote the generation of FOXP3^+^ Tregs. In addition, immediate anaphylactic reactions are decreased in peanut allergic mice and the generation of allergen-specific FOXP3^+^ Tregs are promoted, demonstrating that these immunomodulatory effects take place in both type 1- and type 2-mediated inflammatory diseases. Our findings might open new avenues for novel cannabinoid-based interventions in different inflammatory and immune-mediated diseases.

## Introduction

The immune system protects the host from a broad range of pathogens, while keeping tolerance to self and external innocuous antigens. Alterations of this vital function lead to different inflammatory and immune-mediated diseases^1–4^. Regulatory T cells (Tregs) are a heterogeneous group of T cells with potent suppressive capacity that are broadly classified as: thymus-derived CD25^+^FOXP3^+^ Tregs and peripherally-induced Tregs generated outside the thymus after antigenic stimulation^5^. The generation and maintenance of functional Tregs is indispensable to keep tissue homeostasis and healthy immune responses in many biological contexts such as autoimmunity, metabolic inflammation, pregnancy, cancer, tissue injury, host-commensal interactions, transplantation, acute and chronic infection or allergy^6,7^. Dendritic cells (DCs) are professional antigen-presenting cells linking innate and adaptive immunity^1^. Human circulating DCs are classified as conventional DCs (cDCs) or plasmacytoid DCs (pDCs)^8^. According to their specific phenotype and function, cDCs can be further divided into CD141^+^ type 1 or CD1c^+^ type 2 cDCs. DCs recognise all surrounding antigens, which are then processed and presented to T cells to initiate adaptive immune responses^1,2^. Under non-inflammatory conditions, immature DCs remain tolerogenic and generate Tregs. Mature DCs prime different T cell responses depending on the inflammatory context^1,8,9^. The tolerogenicity of DCs and their ability to prime Tregs is influenced by exogenous signals such as cytokines, metabolites, microbe-derived molecules, or neurotransmitters^4,10–13^.

The human endogenous cannabinoid system (ECS) comprises the endocannabinoid ligands (anandamide and 2-arachidonoylglycerol), the proteins related to their synthesis and degradation and the cannabinoid receptors (CBRs)^14,15^. Endocannabinoids bind to the G protein-coupled receptors (GPCRs) cannabinoid receptor 1 (CB1) and 2 (CB2). Alternative CBRs include other orphan GPCRs (GPCR55, GPCR119 and GPCR18), transient receptor potential cation channels (TRPVs), or nuclear peroxisome proliferator-activated receptors (PPARs)^16–18^. Phytocannabinoids from *Cannabis sativa L*., marijuana, (THC, Δ^9^-tetrahydrocannabinol) and synthetic cannabinoids (HU210, WIN55212-2 or HU308) also activate CBRs and downstream signalling pathways regulating proliferation, differentiation, cell survival, metabolism or immunity^15,19–21^. Different preclinical and clinical studies pointed out cannabinoids as potential therapeutic tools in cancer^15,22^, neurological^23^ and inflammatory diseases^19,21^. Human and mice DCs express functional CBRs^24–26^, but how cannabinoids regulate functional properties of DCs is not yet completely understood. Immunogenicity vs tolerogenicity in DCs is finely regulated by the balance of anabolic vs catabolic metabolism^27–29^. Mammalian target of rapamycin (mTORC1) and AMP kinase (AMPK) are important regulators of anabolic and catabolic metabolism, respectively^30,31^. They also control autophagy, a lysosomal degradation pathway essential for cell survival, differentiation and homeostasis^32,33^. Autophagy might regulate both immunogenicity and tolerogenicity in DCs depending on the physiological context and surrounding microenvironment^33,34^. The activation state of mTORC1 and AMPK is highly sensitive to intracellular and extracellular signals^30,31^. DCs express a plethora of GPCRs, including CBRs, sensing many different substances that might connect the regulation of key metabolic pathways with functional properties^28^. Previous studies reported that cannabinoids regulate cellular respiration and energy metabolism in mouse neurons as essential events in memory processes^14,35^. Cannabinoids might also initiate autophagy leading to apoptosis in cancer cells but not in non-transformed cells^22,36^. Herein, we show for the first time that cannabinoids promote tolerogenic DCs able to prime functional FOXP3^+^ Tregs in the context of inflammatory diseases. Cannabinoids imprint tolerogenicity in human DCs by inducing functional autophagy and subsequent metabolic reprogramming through activation of CB1 and PPARα. In vivo, cannabinoids protects mice against LPS-induced sepsis and peanut-induced anaphylaxis by inducing autophagy and Tregs. The better understanding of the mechanisms by which cannabinoids regulate DC function might well contribute to open alternative avenues for the development of novel cannabinoid-based interventions for different inflammatory and immune-mediated diseases.

## Results

### Cannabinoids promote immature DCs and display anti-inflammatory properties

**Fig. 1:**
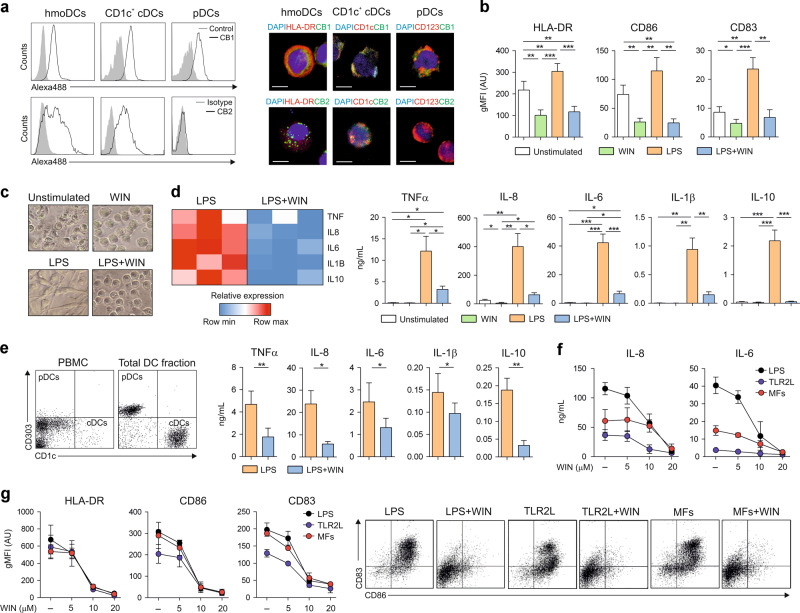
Cannabinoids promote immature and anti-inflammatory human DCs. **a** CB1 and CB2 expression in hmoDCs and purified human blood CD1c^+^ cDCs and pDCs. Representative flow cytometry histograms (left) and confocal images (right). Grey shadowed lines represent the controls and black empty lines represent CB1 or CB2 staining. DAPI (blue), HLA-DR, CD123 or CD1c (red) and CB1 or CB2 (green). White bars, 5 µm. **b** Geometric mean fluorescence intensity (gMFI) of surface markers after stimulation of hmoDCs with medium (unstimulated), WIN55212-2 (WIN, 10 µM), LPS (0.1 µg/mL) or LPS plus WIN55212-2 for 18 h (*n* = 8). **c** Representative images of hmoDC morphology after stimulation with the indicated stimuli. **d** Left, heatmap of cytokine gene expression after stimulation of hmoDCs with LPS or LPS plus WIN55212-2 for 4 h. Right, cytokine production after stimulation of hmoDCs with the indicated stimuli for 18 h (*n* = 8). **e** Left, flow cytometry representative dot plots for pDCs and cDCs in PBMCs and in the total DC fraction. Right, cytokine production after stimulation of total DCs with LPS or LPS plus WIN55212-2 for 18 h (*n* = 6). **f** Suppression of cytokine production and (**g**) gMFI of surface markers after stimulation of hmoDCs with LPS (0.1 µg/mL), TLR2L (25 ng/mL), and maturing factors (MFs: 25 ng/mL IL-1β and 50 ng/mL TNFα) plus different doses of WIN55212-2 for 18 h (*n* = 3). Representative flow cytometry dot plots for CD86 and CD83 expression are shown. Values are mean ± SEM. Statistical significance was determined using One-way Anova (**b**, **d**) or paired *t* test (**e**). *P < 0.05, **P < 0.01, ***P < 0.001.

Human DCs expressed the canonical CBRs as shown by staining purified subsets with HU210-Alexa488, a validated probe to quantify CB1 protein expression^37^, or with anti-CB2 polyclonal antibody. Human monocyte-derived DCs (hmoDCs) and circulating type 2 CD1c^+^ cDCs expressed both CB1 and CB2, whereas plasmacytoid DCs (pDCs) only expressed CB1 (Fig. 1a and Supplementary Fig. 1a, b). Next, we assessed whether synthetic cannabinoids such as WIN55212-2, HU210 (both CB1/CB2 agonist) or HU308 (selective CB2 agonist) could modulate the function of human DCs. WIN55212-2 inhibited in a dose dependent manner TNFα and IL-6 production in LPS-activated hmoDCs without inducing apoptosis or cell death (Supplementary Fig. 2a–c). In contrast, HU210 inhibited IL-6 but not TNFα production and HU308 did not inhibit any of the assayed cytokines (Supplementary Fig. 2a). WIN55212-2 displayed the most potent anti-inflammatory capacity and 10 µM was selected as the optimal effective non-toxic dose for further experiments. WIN55212-2 significantly inhibited the LPS-induced expression of HLA-DR, CD86 and CD83 in hmoDCs (Fig. 1b) and the activation and development of classical long dendrites, promoting immature phenotypes similar to that observed under unstimulated conditions (Fig. 1c). WIN55212-2 alone did not induce cytokines but significantly impaired the production of TNFα, IL-8, IL-6, IL-1β and IL-10 in LPS-stimulated hmoDCs at the mRNA and protein level (Fig. 1d). Supporting these data, cytokine production in total blood DCs containing both cDCs and pDCs after LPS stimulation was also inhibited without affecting cell viability (Fig. 1e and Supplementary Fig. 3). Remarkably, activation of CBRs in hmoDCs abolished the production of IL-8 and IL-6 and the expression of HLA-DR, CD86 and CD83 upon stimulation with TLR2-ligand Pam3CSK4 or with TNFα/IL-1β inflammatory cocktail in a similar dose-dependent manner to that observed for LPS (Fig. 1f, g).

### Generation of tolerogenic DCs via CB1 and PPARα

**Fig. 2:**
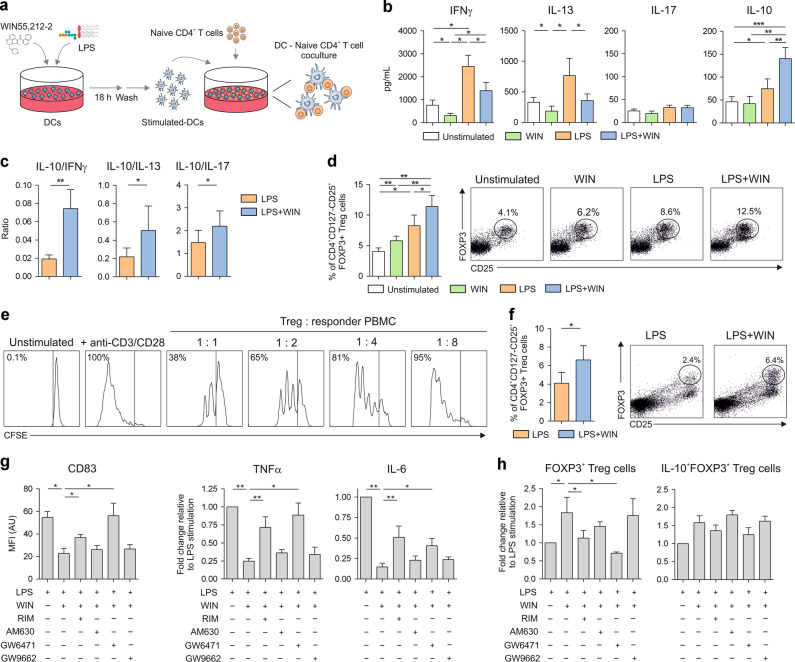
Tolerogenic DCs generated via CB1- and PPARα-mediated activation induce functional FOXP3^+^ Tregs. **a** Scheme of coculture experiments. **b** Cytokines produced by allogeneic naïve CD4^+^ T cells primed by unstimulated, WIN55212-2 (WIN, 10 µM), LPS (0.1 µg/mL) or LPS plus WIN55212-2-treated hmoDCs after 5 days (*n* = 8). **c** Cytokine ratios by primed naïve CD4^+^ T cells in the indicated conditions (*n* = 8). **d** Percentage of induced FOXP3^+^ Tregs under the indicated conditions (*n* = 8). Flow cytometry representative dot plots are shown. **e** Suppression effects of purified induced FOXP3^+^ Tregs generated by LPS/WIN55212-2-stimulated hmoDCs. **f** Percentage of induced FOXP3^+^ Tregs by allogeneic LPS or LPS/WIN55212-2-stimulated total DCs (*n* = 6). Flow cytometry representative dot plots are shown. **g** Geometric mean fluorescence intensity (gMFI) of CD83 expression and TNFα and IL-6 production after stimulation of hmoDCs with LPS (0.1 µg/mL) plus WIN55212-2 (10 µM) in the presence of CB1 (Rimonabant (RIM), 20 µM), CB2 (AM630, 20 µM), PPARα (GW6471, 25 µM) and PPARγ (GW9662, 10 µM) antagonists for 18 h (*n* = 8). **h** Percentage of FOXP3^+^ Tregs generated after 5 days by hmoDCs stimulated with the indicated conditions. Values are mean ± SEM. Statistical significance was determined using One-way Anova (**b**, **d**, **g**, **h**) or Paired *t* test (**c**, **f**). *P < 0.05, **P < 0.01, ***P < 0.001.

To determine whether cannabinoids could condition the capacity of human DCs to polarise T cell responses, we performed allogeneic cocultures (Fig. 2a). Naïve CD4^+^ T cells primed by WIN55212-2-stimulated hmoDCs produced lower levels of IFNγ and IL-13 than those primed by unstimulated hmoDCs (Fig. 2b). Remarkably, hmoDCs activated with LPS/WIN55212-2 generated T cells producing significantly lower levels of IFNγ and IL-13 but significantly higher levels of IL-10 than those primed by LPS-activated hmoDCs (Fig. 2b). IL-17 was low in all the assayed conditions without significant changes (Fig. 2b). The IL-10/IFNγ, IL-10/IL-13 and IL-10/IL-17 ratios were significantly higher when T cells were primed by LPS/WIN55212-2-activated hmoDCs than LPS alone, suggesting the generation of Tregs (Fig. 2c). WIN55212-2-stimulated hmoDCs and LPS/WIN55212-2-activated hmoDCs induced significantly higher numbers of functional FOXP3^+^ Tregs with suppressive capacity than unstimulated or LPS-activated hmoDCs, respectively (Fig. 2d, e). Supporting these data, LPS/WIN55212-2-activated total blood DCs also induced significantly higher numbers of FOXP3^+^ Tregs than LPS alone (Fig. 2f). Previous data showed that WIN55212-2 effects might be mediated not only by canonical CB1/CB2 but also by other alternative CBRs such as PPARα or PPARγ^38,39^. HmoDCs expressed PPARα and PPARγ at the mRNA and protein level (Supplementary Fig. 3). The pharmacological blockade of CB1 (Rimonabant) or PPARα (GW6471) impaired the inhibitory effects of WIN55212-2 on CD83 expression, TNFα and IL-6 production in LPS-activated hmoDCs, whereas the blockade of CB2 (AM630) or PPARγ (GW9662) did not (Fig. 2g). Similarly, CB1 or PPARα antagonists impaired the capacity of LPS/WIN55212-2-stimulated hmoDCs to generate FOXP3^+^ Tregs as well as IL-10-producing FOXP3^+^ Tregs (Fig. 2h and Supplementary Fig. 5), demonstrating that CB1 and PPARα are the main receptors involved in the generation of human tolerogenic DCs.

### Cannabinoids induce functional autophagy and metabolic rewiring in human DCs

**Fig. 3:**
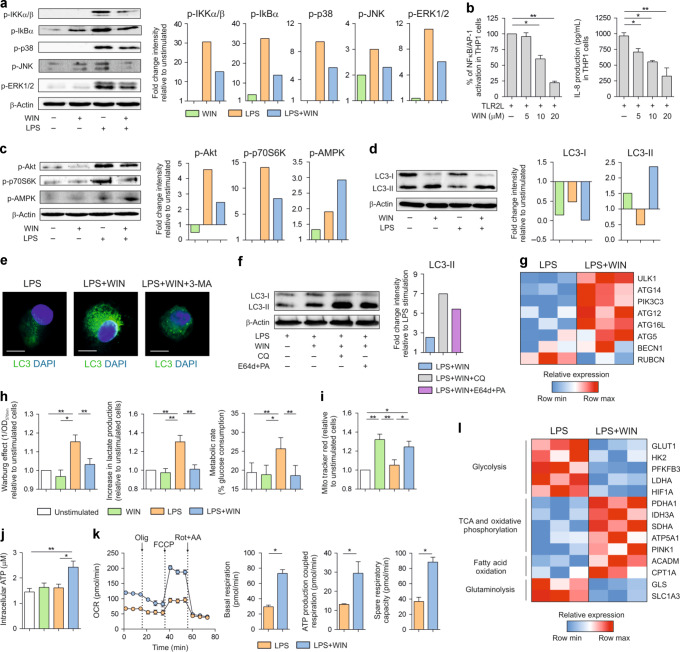
Triggering of CBRs inhibits inflammatory signalling pathways and promotes autophagy and metabolic rewiring in human DCs. **a** Left, western blot of protein extracts from hmoDCs stimulated for 15 min in the indicated conditions. Right, quantification of the reactive phosphorylated bands by scanning densitometry. **b** TLR2L-induced NFκB/AP-1 activation and IL-8 production by THP1-XBlue^TM^ cells with different doses of WIN55212-2 (WIN) for 18 h (*n* = 5). **c** Left, western blot of hmoDCs stimulated for 30 min in the indicated conditions. Right, quantification of the reactive phosphorylated bands by scanning densitometry. **d** Left, western blot of hmoDCs stimulated for 18 h in the indicated conditions. Right, quantification of the reactive bands by scanning densitometry. **e** Representative confocal images of LC3 staining of hmoDCs stimulated for 18 h in the indicated conditions. LC3 (green), DAPI (blue). White bars, 5 µm. **f** Left, western blot of hmoDCs preincubated with chloroquine (CQ) or E64d plus pepstatin A (PA) and stimulated for 18 h with LPS plus WIN55212-2. Right, quantification of the reactive bands by scanning densitometry. **g** Heatmap of autophagy-related gene expression after stimulation of hmoDCs with LPS or LPS/WIN55212-2 for 4 h. **h** Quantification of the induced Warburg effect and lactate content in cell-free supernatants relative to unstimulated hmoDCs and glucose consumption determined as metabolic rate of the indicated conditions (*n* = 8). **i** Fluorescence intensity of stimulated hmoDCs stained with Mito Tracker Red (*n* = 8). **j** Intracellular ATP levels in hmoDCs after 18 h of stimulation with the indicated conditions (*n* = 6). **k** Kinetic study of mitochondrial OCR in LPS or LPS/WIN55212-2-stimulated hmoDCs by sequential addition of oligomycin (Olig), FCCP, and rotenone/antimycin A (Rot + AA). Quantification of basal respiration, ATP production coupled respiration and spare respiratory capacity of hmoDCs are included (*n* = 6 of two independent experiments). **l** Heatmap of metabolism-related gene expression after stimulation of hmoDCs with LPS or LPS/WIN55212-2 for 4 h. Values are mean ± SEM. Statistical significance was determined using One-way Anova. *P < 0.05, **P < 0.01. p-IKKα/β, phosphorylated-IκB kinase subunits alpha and beta; p-IκB, phosphorylated-inhibitor of NFκB; p-p38, phosphorylated-p38 kinase; p-JNK, phosphorylated-c-Jun N-terminal kinase; p-ERK, phosphorylated-extracellular signal regulated kinase; NFκB, nuclear factor κB; AP-1, activation protein 1; p-Akt, phosphorylated-protein kinase B; p-p70S6K, phosphorylated-p70S6 kinase; p-AMPK, phosphorylated-AMP-activated protein kinase; LC3-I or II, microtubule-associated protein 1 A/1B-light chain 3-I or II; 3-MA, 3-methyladenine; ULK1, Unc-51 like kinase; ATG5 12, 14 or 16 L, autophagy-related gene 5, 14 or 16 L; PIK3C3, phosphatidylinositol 3-kinase catalytic subunit type 3; BECN1, Beclin; RUBCN, Rubicon; GLUT1, glucose transporter 1; HK2, hexokinase 2; PFKFB3, phosphofructokinase 3; LDHA lactate dehydrogenase, HIF1A hypoxia-inducible factor 1 alpha, PDHA pyruvate dehydrogenase, IDH3A isocitrate dehydrogenase, SDHA succinate dehydrogenase, ATP5A1 ATP synthase subunit alpha, PINK1 PTEN-induced kinase 1, ACADM acyl-coenzyme A dehydrogenase, CPT1A carnitine O-palmitoyltransferase 1, GLS glutaminase, SLC1A3 amino acid transporter 1.

To assess molecular mechanisms underlying cannabinoids immunomodulatory effects, we initially studied different inflammatory signalling pathways activated by LPS in human DCs. WIN55212-2 inhibited LPS-induced phosphorylation of IKKα/β, IκBα, p38, JNK and ERK1/2, impairing the activation of NF-κB and MAPK signalling pathways (Fig. 3a). Supporting these data, TLR2L-induced NFκB/AP-1 activation and IL-8 production in THP1-XBlue^TM^, a monocytic cell line expressing CB1 and CB2 (Supplementary Fig. 4a, b) was also inhibited in a dose-dependent manner. (Fig. 3b). In addition, WIN55212-2 inhibited the LPS-induced phosphorylation of Akt and p70S6K, demonstrating its capacity to impair LPS-induced mTOR activation in hmoDCs (Fig. 3c). Remarkably, phosphorylation of AMPK in LPS-activated hmoDCs was enhanced, supporting the anti-inflammatory effects and suggesting autophagy induction (Fig. 3c). To further investigate the capacity of cannabinoids to promote autophagy in human DCs, we monitored changes in LC3 isoforms. The levels of LC3-II, the lipidated and autophagosome-associated form of LC3, were increased by WIN55212-2 and decreased by LPS in hmoDCs (Fig. 3d). LPS/WIN55212-2-stimulated hmoDCs displayed higher levels of LC3-II than LPS-activated hmoDCs (Fig. 3d), demonstrating the capacity to promote autophagy. Higher levels of LC3-II associated to autophagosomes were also demonstrated in LPS/WIN55212-2-stimulated hmoDCs by confocal microscopy, which was impaired by pharmacological autophagy inhibition (Fig. 3e). Co-incubation with chloroquine or the lysosomal protease inhibitors E64d and pepstatin A, which blocks the last steps of autophagy degradation, enhanced WIN55212-2-induced accumulation of LC3-II (Fig. 3f), confirming that WIN55212-2 induces a dynamic autophagy flux in hmoDCs. In addition, LPS/WIN55212-2-stimulated hmoDCs displayed higher mRNA levels of the autophagy related-genes ULK1, ATG14, PIK3C3, ATG12, ATG16L and ATG5 and lower levels of Rubicon (RUBCN), an inhibitor of canonical autophagy and exclusive protein of LC3-associated phagocytosis (LAP), than LPS-activated hmoDCs (Fig. 3g).

Next, we wanted to further investigate the potential capacity of cannabinoids to rewire the metabolic signature imprinted by LPS in human DCs. WIN55212-2 significantly inhibited LPS-induced Warburg effect, lactate production and metabolic rate in hmoDCs (Fig. 3h). In contrast, mitochondrial membrane potential (Fig. 3i) and intracellular ATP production were significantly enhanced in LPS/WIN55212-2-stimulated hmoDCs (Fig. 3j), indicating that cannabinoids rewire the glucose metabolism of LPS-activated hmoDCs towards decreased glycolysis and enhanced mitochondrial activity and oxidative phosphorylation. To confirm these data, we examined real-time oxygen consumption rate (OCR) using a Seahorse bioanalyzer. Basal respiration, maximal respiration, ATP production coupled to respiration and spare respiratory capacity (SCR) were significantly increased in LPS/WIN55212-2-stimulated hmoDCs than LPS-stimulated hmoDCs (Fig. 3k). Supporting these data, the mRNA levels of the glycolysis-related genes GLUT1, HK2, PFKFB3, LDHA and HIF1A were downregulated by WIN55212-2 in LPS-activated hmoDCs whereas enzymes involved in the tricarboxilic acid (TCA) cycle or in oxidative phosphorylation such as PDHA1, IDH3A, SDHA, ATP5A1 and PINK1 were upregulated (Fig. 3l). The mRNA levels of proteins involved in fatty acid oxidation, such as ACADM or CPT1A, were upregulated in LPS/WIN55212-2-stimulated hmoDCs whereas enzymes involved in glutaminolysis, such as GLS or SLC1A3, were downregulated (Fig. 3l), suggesting that fatty acids but not glutamine fuel the TCA in LPS/WIN55212-2-stimulated hmoDCs (Fig. 3l). No changes in genes related to fatty acid synthesis, pentose phosphate pathway or other TCA cycle genes were observed (Supplementary Fig. 5).

### CB1- and PPAR-α-induced autophagy drives metabolic reprogramming and the generation of human tolerogenic DCs

**Fig. 4:**
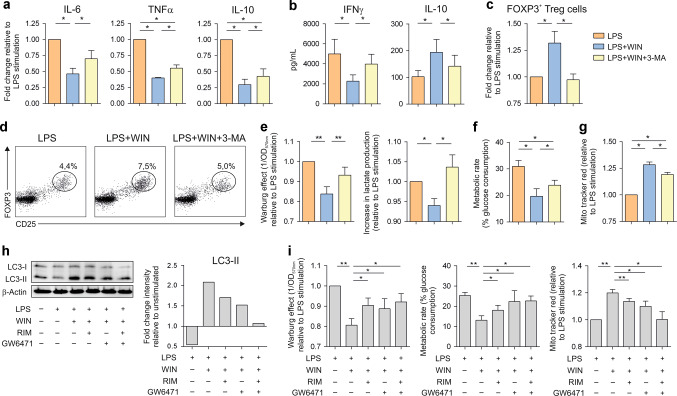
CBRs-induced autophagy drives metabolic reprogramming and human tolerogenic DCs generation. **a** Gene expression levels of hmoDCs stimulated with LPS (0.1 µg/mL) plus WIN55212-2 (WIN, 10 µM) or in the presence of the autophagy inhibitor 3-methyladenine (3-MA, 25 µM) relative to LPS-stimulated condition (*n* = 5). **b** Cytokines produced by naïve CD4^+^ T cells primed by hmoDCs stimulated in the indicated conditions after 5 days (*n* = 6). **c** Increment in FOXP3^+^ Tregs generation relative to LPS-stimulation condition. **d** Flow cytometry representative dot plots. **e** Quantification of the induced Warburg effect and lactate content in cell-free supernatants relative to LPS stimulation (*n* = 6). **f** Glucose consumption determined as metabolic rate of the indicated conditions (*n* = 6). **g** Fluorescence intensity of hmoDCs stained with Mito Tracker Red relative to LPS-stimulated condition (*n* = 6). **h** Left, western blot of hmoDCs stimulated with LPS, LPS/WIN55212-2 or LPS/WIN55212-2 in the presence of CB1 (Rimonabant, (RIM), 10 µM) and PPARα (GW6471, 25 µM) antagonists for 18 h. Right, quantification of the reactive bands by scanning densitometry. **i** Quantification of the induced Warburg effect, metabolic rate and fluorescence intensity of Mito Tracker Red stained cells in hmoDCs relative to LPS stimulation (*n* = 6). Values are mean ± SEM. Statistical significance was determined using One-way Anova. *P < 0.05, **P < 0.01.

Autophagy inhibition in LPS/WIN55212-2-stimulated hmoDCs significantly increased the mRNA expression levels of IL-6, TNFα and IL-10 (Fig. 4a) and the levels of IFNγ produced by primed T cells (Fig. 4b) whereas significantly impaired the production of IL-10 by T cells and the generation of FOXP3^+^ Tregs (Fig. 4b–d), demonstrating that cannabinoids-induced autophagy is indispensable to generate tolerogenic human DCs with capacity to prime Tregs. Pharmacological blocking of autophagy in LPS/WIN55212-2-stimulated hmoDCs significantly increased the Warburg effect, lactate production and metabolic rate (Fig. 4e, f) and reduced mitochondrial membrane potential (Fig. 4g), demonstrating that autophagy is driving the metabolic reprogramming induced in LPS/ WIN55212-2-stimulated DCs. Simultaneous inhibition of CB1 and PPARα impaired autophagy induction in hmoDCs as demonstrated by the restoration of LC3-II to basal levels (Fig. 4h) and inhibited metabolic rewiring, including the Warburg effect and metabolic rate reduction and the increase of mitochondrial membrane potential in LPS/ WIN55212-2-stimulated hmoDCs (Fig. 4i). Collectively, these data showed that CB1- and PPARα-mediated signalling pathways promote functional autophagy, which is essential to drive metabolic reprogramming and the subsequent generation of human tolerogenic DCs with the capacity to prime Tregs.

### Triggering of CBRs confers protection against LPS-induced sepsis via autophagy and promotes the generation of functional Tregs

**Fig. 5:**
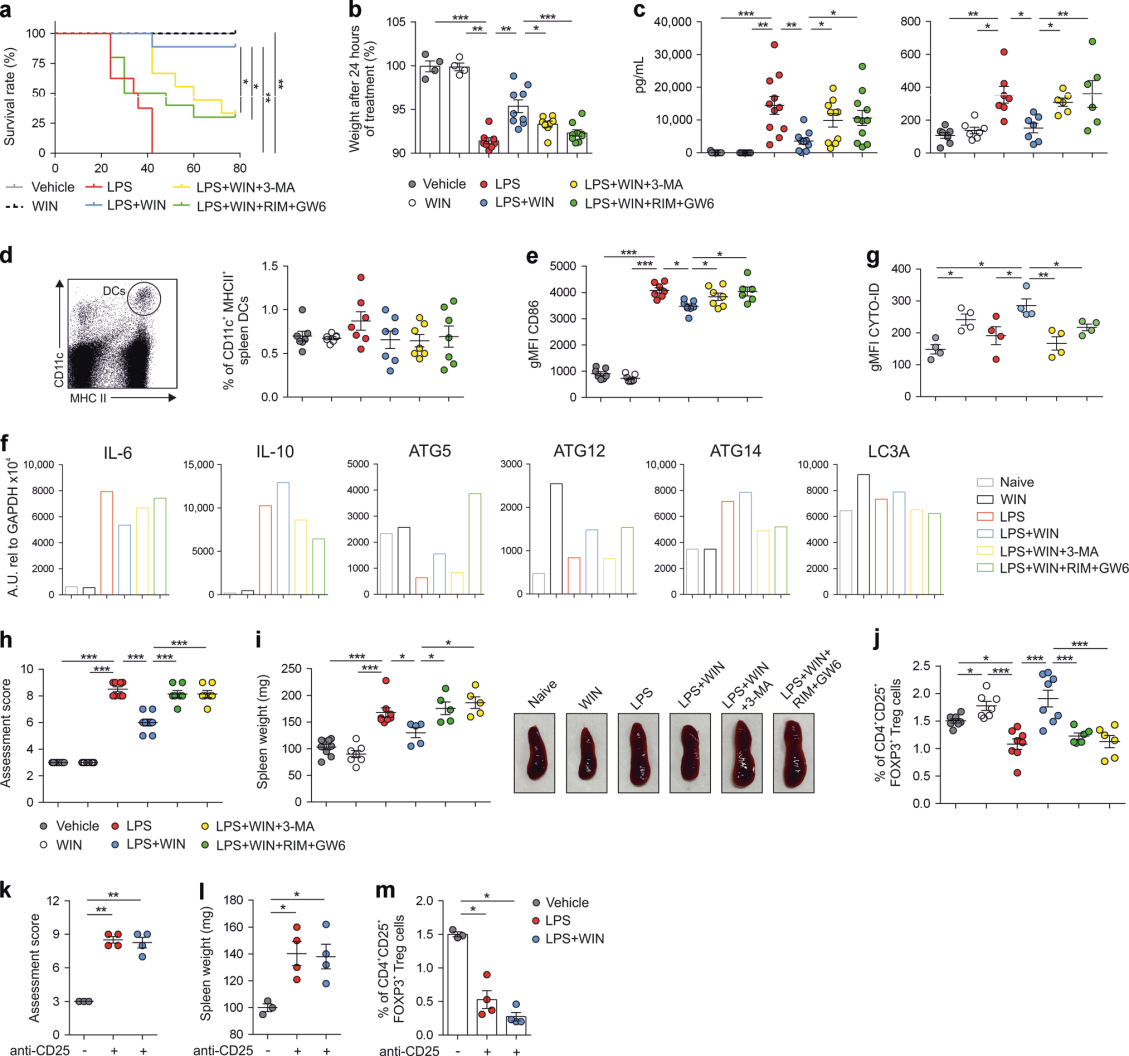
Cannabinoids protect against LPS-induced sepsis in BALB/c mice. **a** Survival rate of mice treated with WIN55212-2 (WIN, 5 mg/kg), LPS (20 mg/kg), LPS/WIN55212-2 and LPS/WIN55212-2 in the presence of the autophagy inhibitor 3-MA (15 mg/kg) or CB1 (Rimonabant (RIM), 5 mg/kg) and PPARα (GW6471 (GW6), 5 mg/kg) antagonists monitored for 78 h (*n* = 10 of two independent experiments). **b** Body weight after 24 h of the indicated treatment (*n* = 4–9 of two independent experiments). **c** Serum and bronchoalveolar lavage fluid (BALF) IL-6 levels after 16 h of the indicated treatment (*n* = 6–11 of two independent experiments). **d** Left, flow cytometry representative dot plots of spleen DCs. Right, percentage of spleen CD11c^+^ MHCII^+^ DCs from mice treated with the indicated conditions for 16 h (*n* = 7–8 of two independent experiments). **e** Geometric mean fluorescence intensity (gMFI) of CD86 expression in spleen CD11c^+^ MHCII ^+^ DCs from mice treated with the indicated conditions for 16 h. **f** mRNA expression levels of the cytokines IL-6 and IL-10 and autophagy-related genes ATG5, ATG12, AG14 and LC3A in isolated spleen DCs from mice treated with the indicated conditions. Data were obtained from a pull of spleen DCs from four mice treated with the same stimuli. One representative example of two independent experiments is shown. **g** gMFI of CYTO-ID green dye in spleen DCs from mice treated with the indicated conditions (*n* = 4 of two independent experiments). **h** Assessment score of physical appearance 3 days after the treatment with WIN55212-2 (5 mg/kg), LPS (10 mg/kg), LPS/WIN55212-2 and LPS/WIN55212-2 in the presence of the autophagy inhibitor 3-MA (15 mg/kg) or CB1 (Rimonabant (RIM), 5 mg/kg) and PPARα (GW6471 (GW6) 5 mg/kg) antagonists (*n* = 7 of two independent experiments). **i** Left, spleen weight from mice treated with the indicated conditions after 3 days (*n* = 5–7 of two independent experiments). Right, representative examples of spleen sizes. **j** Percentage of FOXP3^+^ Tregs in spleen from mice treated with the indicated stimuli (*n* = 6–8 of two independent experiments). **k** Assessment score of physical appearance 3 days after the treatment with LPS (10 mg/kg) and LPS/WIN55212-2 (WIN, 5 mg/kg) in Treg depleted mice with anti-CD25 antibody (*n* = 4 of one independent experiment). **l** Spleen weight from Treg depleted mice treated with the indicated conditions after 3 days (*n* = 4 of one independent experiment). **m** Percentage of FOXP3^+^ Tregs in spleen from Treg depleted mice treated with the indicated stimuli (*n* = 4 of one independent experiment). Values are mean ± SEM. Statistical significance was determined using One-way Anova. *P < 0.05, **P < 0.01, ***P < 0.001.

To assess cannabinoids immunomodulatory effects in vivo we initially used a LPS-induced sepsis model. Mice treated with lethal doses of LPS died after 42 h whereas those treated with vehicle or WIN55212-2 alone survived for the 78 h of follow up (Fig. 5a). The administration of LPS in the presence of WIN55212-2 significantly increased the survival rate (up to 90%) at 78 h, which was significantly impaired by pharmacological inhibition of autophagy or CB1/PPARα (Fig. 5a). LPS-treated mice showed significant weight loss compared to vehicle- or WIN55212-2-treated mice after 24 h (Fig. 5b). The weight loss of LPS/WIN55212-2-treated mice was less pronounced than LPS-treated mice, which was impaired by blocking autophagy or CB1/PPARα (Fig. 5b). The levels of IL-6 in serum and bronchoalveolar lavage fluid were increased in LPS-treated mice after 16 h, which were significantly decreased in the presence of WIN55212-2 and restored to the levels observed in LPS-treated mice upon autophagy inhibition or CB1/PPARα blockade (Fig. 5c). Next, we quantified total spleen CD11c^+^MHCII^+^ DCs without significant differences among the assayed conditions (Fig. 5d). However, spleen DCs from LPS-treated mice displayed a more mature phenotype than those from vehicle- or WIN55212-2-treated mice as determined by the higher CD86 expression, which was reduced in DCs from LPS/WIN55212-2-treated mice (Fig. 5e). Inhibition of autophagy or CB1/PPARα in LPS/WIN55212-2-treated mice increased CD86 levels in spleen DCs (Fig. 5e). Purified total spleen DCs from LPS/WIN55212-2-treated mice expressed less IL-6 and more IL-10 mRNA than those from LPS-treated mice, which were restored upon autophagy or CB1/PPARα inhibition (Fig. 5f). ATG5, ATG12 and LC3A mRNA levels were higher in purified total spleen DCs from WIN55212-2- than vehicle-treated mice (Fig. 5f). Similarly, DCs from LPS/WIN55212-2-treated mice showed increased ATG5, ATG12, ATG14 and LC3A mRNA levels than those from LPS-treated mice (Fig. 5f), which were reduced when inhibiting autophagy. CB1/PPARα blockade reduced ATG14 and LC3A, but not ATG12 or LC3A mRNA levels (Fig. 5f). The capacity of WIN55212-2 to induce autophagy in spleen DCs under LPS sepsis conditions was further confirmed by flow cytometry using CYTO-ID (Fig. 5g).

Next, we investigated the capacity of WIN55212-induced tolerogenic DCs to generate Tregs in vivo. Mice treated with non-lethal doses of LPS developed clinical symptoms after 3 days as scored by the appearance of coat, activity and posture, which were significantly reduced in LPS/WIN55212-2-treated mice and restored upon inhibition of autophagy or CB1/PPARα (Fig. 5h). Non-lethal doses of LPS induced strong splenomegaly that was impaired by WIN55212-2 through mechanisms depending on autophagy, CB1 and PPARα (Fig. 5i). Mice treated with WIN55212-2 alone showed significantly higher percentages of spleen FOXP3^+^ Tregs than vehicle-treated mice (Fig. 5j). Remarkably, LPS significantly reduced the frequency of spleen FOXP3^+^ Tregs, which was significantly higher in LPS/WIN55212-2- than LPS-treated mice (Fig. 5j). Autophagy inhibition or the blockade of CB1/PPARα in LPS/WIN55212-2-treated mice significantly reduced the percentage of spleen FOXP3^+^ Tregs to the levels observed in LPS-treated mice (Fig. 5j). To further assess the functional role of Tregs in this model, we depleted them with anti-CD25 antibody (Supplementary Fig. 6). WIN55212-2 did not reduce the clinical symptoms and the increase of spleen weight induced by non-lethal doses of LPS in Treg depleted mice (Fig. 5k, l), highlighting the important contribution of Tregs in the WIN55212-2-induced clinical recovery and inflammation resolution upon LPS exposure. Supporting these data, we did not observed an increase in spleen FOXP3^+^ Tregs after LPS/WIN55212-2 administration in Treg depleted mice (Fig. 5m). Collectively, our in vivo data demonstrate that cannabinoids confer mice protection in an LPS-induced sepsis model by impairing lethal acute inflammation via CB1- and PPARα-mediated autophagy induction and promote tolerogenic DCs able to prime functional Tregs that contribute to clinical recovery and homeostasis.

### Cannabinoids decrease anaphylaxis in peanut sensitised mice and promote the generation of peanut-specific Tregs

**Fig. 6:**
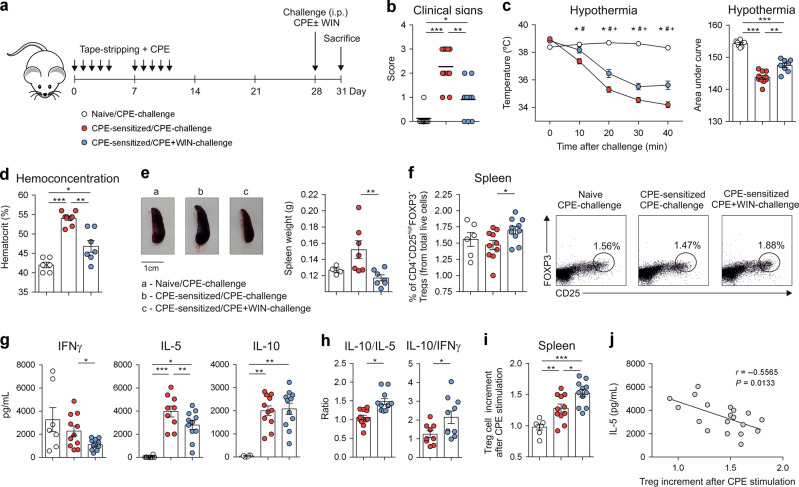
Cannabinoids reduce anaphylaxis in peanut sensitised BALB/c mice. **a** Scheme of the epicutaneous sensitisation protocol; i.p., intraperitoneally. **b** Clinical signs observed after challenge with crude peanut extract (CPE, 2.5 g/mouse) or CPE plus WIN55212-2 (WIN, 20 µg/mouse). **c** Left, changes in body temperature 40 min after challenge. *Naïve vs CPE-Sensitised/CPE Challenge, #CPE-Sensitised/CPE Challenge vs CPE-Sensitised/CPE + WIN55212-2 Challenge, + Naïve vs CPE-Sensitised/CPE + WIN55212-2 Challenge. Right, area under the curve (temperature vs time) of the indicated conditions. **d** Hemoconcentration 40 min after challenge. **e** Left, one representative example of spleen size and right, spleen weight 72 h after challenge. **f** Left, percentage of spleen FOXP3^+^ Tregs in the indicated conditions. Right, Flow cytometry representative dot plots of generated splenic FOXP3^+^ Tregs. **g** Cytokine production and (**h**) cytokine ratios by splenocytes from the indicated mice, stimulated in vitro with CPE (250 µg/mL) for 4 days. **i** Increment of FOXP3^+^ Tregs generation after in vitro CPE stimulation for 4 days relative to unstimulated condition. **j** Negative correlation of increased Tregs with IL-5 levels produced by CPE-stimulated splenocytes. Values are the mean ± SEM. *n* = 5–10 of two independent experiments. Statistical significance was determined using One-way Anova and Spearman test (**j**): *P < 0.05, **P < 0.01 and ***P < 0.001.

To assess whether the immunomodulatory effects of cannabinoids could be also clinically relevant in the context of type 2-mediated inflammatory diseases, we used a model of peanut-induced anaphylaxis, a severe allergic reaction characterised by multiorgan dysfunction and potentially fatal outcome. Mice were epicutaneously sensitised to peanut using crude peanut extract (CPE) for 2 weeks (Fig. 6a) and allergic sensitisation was confirmed by quantifying serum peanut-specific IgE and IgG_1_ antibodies 10 days after the last sensitisation (Supplementary Fig. 7). CPE-sensitised mice were intraperitoneally challenged with CPE alone or CPE plus WIN55212-2 at day 28 (Fig. 6a). Peanut sensitised mice challenged with CPE in combination with WIN55212-2 exhibited significantly lower clinical symptoms, scored as hind leg scratching in ear canal, reduced movement, puffy eyes and convulsion, than sensitised mice challenged with CPE alone (Fig. 6b). Sensitised mice challenged with CPE/WIN55212-2 experienced a significantly lower drop in body temperature, a surrogate marker of the severity of systemic anaphylaxis, than those challenged with CPE alone (Fig. 6c). WIN55212-2 also impaired the CPE-induced increase of hematocrit associated to edema (Fig. 6d). Collectively, these data revealed that WIN55212-2 decreases CPE-induced anaphylaxis and vascular leakage in peanut sensitised mice. We also studied WIN55212-2 effects in late-phase allergic responses. After 72 h of the challenge, CPE-sensitised mice challenged with CPE alone presented significant splenomegaly compared to naïve/CPE-challenged mice (Fig. 6e). Spleen weight of CPE-sensitised mice challenged with CPE plus WIN55212-2 was significantly reduced compared to CPE-challenged mice (Fig. 6e). Interestingly, the percentage of spleen FOXP3^+^ Tregs was significantly higher in CPE/WIN55212-2-challenged than CPE-challenged CPE-sensitised mice (Fig. 6f), suggesting that WIN55212-2 might well contribute to the regulation of CPE-mediated late-phase responses in sensitised mice. To assess allergen-specific responses, splenocytes from CPE-challenge naïve mice, CPE-challenge or CPE/WIN55212-2-challenge CPE-sensitised mice were in vitro stimulated with CPE. Splenocytes from CPE-challenged sensitised mice produced significantly higher levels of IL-5 and IL-10 than those from naïve mice (Fig. 6g). In contrast, in vitro CPE-stimulated splenocytes from CPE-sensitised and CPE/WIN55212-2-challenged mice produced lower levels of IFNγ and IL-5 than CPE-challenged mice without significant changes in IL-10 (Fig. 6g). The IL-10/IL-5 and IL-10/IFNγ ratios were significantly higher in splenocytes from CPE-sensitised mice challenged with CPE/WIN55212-2 than CPE alone, suggesting the induction of CPE-specific tolerogenic T cell responses (Fig. 6h). The increment in FOXP3^+^ Tregs after in vitro CPE stimulation was significantly higher in splenocytes from CPE/WIN55212-2- than CPE-challenged mice (Fig. 6i). This Tregs increment was negatively correlated with IL-5, highlighting that Tregs induction is associated with lower Th2 responses (Fig. 6j). These data support the ability of WIN55212-2 to immunomodulate food-induced allergic responses, demonstrating the potential immunomodulatory capacity of cannabinoids in both type 1- and type 2-mediated inflammatory diseases.

## Discussion

In this study, we uncover unprecedented underlying molecular mechanisms by which cannabinoids promotes tolerogenic DCs able to prime functional FOXP3^+^ Tregs in the context of different inflammatory diseases. Under inflammatory conditions, cannabinoids imprints tolerogenicity in human DCs by inhibiting NF-κB, MAPK and mTOR signalling pathways while inducing AMPK and functional autophagy via CB1- and PPARα-mediated activation. CBRs-induced autophagy drives subsequent metabolic rewiring that shifts glucose metabolism from glycolysis and Warburg effect towards increased mitochondrial activity and oxidative phosphorylation, promoting the generation of human tolerogenic DCs able to polarise functional IL-10-producing FOXP3^+^ Tregs. The synthetic cannabinoid WIN55212-2 exhibits in vivo protective and anti-inflammatory effects in LPS-induced sepsis by mechanisms depending on CB1- and PPARα-mediated autophagy induction and also promotes the generation of FOXP3^+^ Tregs. In addition, WIN55212-2 decreases immediate anaphylactic reactions in peanut allergic mice and promotes the generation of allergen-specific FOXP3^+^ Tregs, indicating that cannabinoids display immunomodulatory effects in both type 1- and type 2-mediated inflammatory diseases. Our findings might well contribute to open future avenues for the development of novel cannabinoid-based interventions for different inflammatory and immune-mediated diseases.

Due to its indispensable role in the regulation of immune responses, DCs are regarded as ideal targets for therapeutic interventions^40,41^. Although data in human are still scarce, DCs express all the components of the ECS^24^. Here, we confirmed the expression of CBRs in different human blood DC subsets, including hmoDCs, type 2 cDCs and pDCs. Some studies investigated the role of cannabinoids in the control of mice DC function, but studies assessing the capacity of cannabinoids to modulate the function of human DCs are comparatively rare. Roth et al. showed that the exposure of human monocytes to THC alters their differentiation into DCs, reducing their activation capacity^42^. THC and CB2 agonists also reduce the production of cytokines in human pDCs^43,44^. Here, we showed that several synthetic cannabinoids might exert immunomodulatory effects on human DCs and identified WIN55212-2 as the most potent. WIN55212-2 significantly impairs the expression of HLA-DR, CD86/CD83 and cytokine production in LPS-stimulated hmoDCs, promoting immature and anti-inflammatory phenotypes. Remarkably, this capacity was not exclusively restricted to LPS activation and it was also observed after stimulation with other stimulus such as TLR2L Pam3CSK4 or pro-inflammatory cytokines TNFα/IL-1β. WIN55212-2 impairs the polarisation of effector T cells induced by LPS-activated human DCs and increases the generation of functional IL-10-producing FOXP3^+^ Tregs. Although WIN55212-2 is a potent non-selective CB1/CB2 agonist, it might also exert its anti-inflammatory effects through other alternative CBRs^18,45^. CB2 selective agonists are immunosuppressive in a variety of induced autoimmune diseases in mice^46^. At this regard, a CB2 selective agonist induced Tregs and IL-10 in mouse spleen cell cultures, which suppressed mixed lymphocyte reactions in vitro^47^. Although the functional role of CB1 in immune cells is less studied, especially in humans, WIN55212-2 ameliorates multiple sclerosis in mice by reducing proinflammatory mediators in a CB1-dependent manner^48^. Interestingly, here we showed, for the first time, that WIN55212-2 imprints tolerogenic human DCs able to prime functional IL-10-producing FOXP3^+^ Treg cells by mechanisms mainly depending on the activation of CB1 and PPARα.

The molecular mechanisms by which cannabinoids might control DCs specifications remain poorly understood. Previous studies showed the cross-talk between TLR- and CBR-mediated signalling pathways in monocytes, macrophages and microglia^49–51^. WIN55212-2 inhibits NF-κB and MAPK signalling pathways induced not only by LPS in human DCs, but also by Pam3CSK4 in THP1 cells. Interestingly, CBRs-activation inhibits the LPS-induced activation of mTOR and induces AMPK activation, a key regulator of autophagy. Salazar et al. showed that THC promotes autophagy-mediated cell death in human and mice cancer cells^36^. Similarly, THC and synthetic cannabinoids induce AMPK-dependent autophagy in hepatocellular carcinoma and pancreatic cancer cells^52,53^. Here, we show that WIN55212-2 activates canonical autophagy flux in human DCs, which drives metabolic reprogramming and the generation of human tolerogenic DCs able to polarise functional IL-10-producing FOXP3^+^ Tregs. Tomic et al. found that graphene quantum dots suppresses pro-inflammatory T cell responses through the autophagy-dependent induction of tolerogenic human DCs^54^. Supporting these data, mice with DCs lacking autophagy displayed Tregs with reduced suppressive function and stability^34^. mTOR promotes anabolic processes for the synthesis of macromolecules needed for cell growth, proliferation and activation^55–57^. In contrast, AMPK inhibits anabolism and activate catabolic processes for ATP production^55,58^. Metabolic reprogramming plays a significant role in the control of DCs function by regulating tolerogenicity vs immunogenicity^9,28^. Whether cannabinoids could regulate key metabolic events in human DCs and their functional consequences remained elusive. Here, we showed that LPS-activated hmoDCs display a high rate of glycolysis and lactic acid fermentation (Warburg effect)^59,60^, which is fully abolished by WIN55212-2. Consistent with our results, Malinarich et al. reported that LPS-maturated hmoDCs are more glycolytic than immature hmoDCs, whereas tolerogenic hmoDCs display less glycolytic capacity than mature DCs^59^. Carnosol and curcumin also attenuate the increase in glycolysis in LPS-activated human DCs by the activation of AMPK, which is necessary for the immunosuppressive properties of these polyphenols^61^. Tolerogenic DCs have been reported to possess a greater capacity for oxidative phosphorylation and fatty acid oxidation^62^. For example, tolerogenic hmoDCs generated by treatment with dexamethasone or vitamin D3 exhibit enhanced catabolism and metabolic plasticity and increased expression of genes involved in oxidative phosphorylation and fatty acid oxidation^59,63^. Our real-time functional metabolic experiments showed that WIN55212-2 favours oxygen consumption and mitochondrial activity, which was accompanied by the increased expression of genes involved in oxidative phosphorylation and fatty acid oxidation together with decreased expression of genes involved in glycolysis in LPS-activated hmoDCs. At this regard, Dando et al. described that the CB1 agonist arachidonoyl cyclopropamide inhibits the glycolytic pathway and induces AMPK-dependent autophagy in pancreatic cells^52^. PPARα agonists also induce autophagy and enhance mitochondrial respiration in macrophages during mycobacterial infection^64^.

We assessed the potential in vivo relevance of our findings in a preclinical LPS-induced sepsis model, characterised by local and systemic inflammatory responses^65^. Cannabinoids exert in vivo protective and anti-inflammatory effects in LPS-induced sepsis by mechanisms that rely on CB1- and PPARα-mediated autophagy induction. Jiao et al. also described that PPARα activation reduces LPS-induced acute liver damage by promoting autophagy^66^. Supporting our in vitro data, WIN55212-2 imprints tolerogenic properties in spleen DCs and promotes the generation of functional Tregs by mechanisms depending on autophagy induction in LPS-induced septic mice, which significantly contributed to clinical recovery, resolution of inflammation and homeostasis. Previous studies reported that Tregs together with IL-10 are essential for the survival of septic mice and the control of excessive immune responses, avoid deleterious consequences for the tissues and contribute to pathogen clearance by keeping homeostasis^67,68^. However, if the immunosuppression induced by Tregs persists, the susceptibility to secondary infections might increase. Therefore, further research investigating the immunomodulatory capacity of cannabinoids in sepsis is warranted. Remarkably, we confirmed cannabinoids immunoregulatory properties in a peanut allergy model, which is the most common cause of fatal food-induced anaphylactic reactions^69,70^. Allergen-specific Tregs are essential in the induction and maintenance of allergen tolerance in healthy responses and successful allergen-specific immunotherapy^69,71^. Our in vivo data showed that the administration of WIN55212-2 during peanut challenge in allergic mice decreases the immediate anaphylactic reaction and promotes the generation of allergen-specific Tregs that are associated with lower Th2 responses in chronic late-phase responses, demonstrating the potential immunomodulatory capacity of cannabinoids in both type 1- and type 2-mediated inflammatory diseases.

In summary, we uncover underlying molecular mechanisms showing that CB1- and PPARα-mediated autophagy induction drives metabolic rewiring in DCs that shifts glucose metabolism from glycolysis and Warburg effect towards increased mitochondrial activity and oxidative phosphorylation, promoting the generation of tolerogenic DCs able to polarise functional FOXP3^+^ Tregs. We also demonstrated the potential clinical applications of these findings in two different in vivo inflammatory models such as sepsis and peanut allergy. Our results might well contribute to pave the way for the future development of novel cannabinoid-based strategies for different inflammatory and immune-mediated diseases.

## Methods

### Material, media and reagents

We used RPMI 1640 medium (Lonza) supplemented (cRPMI) with 10% heat‐inactivated fetal bovine serum (FBS, Hyclone) 100 μg/mL normocin (InvivoGen), 50 μg/mL penicillin‐streptomycin, 1% nonessential amino acids, 1% MEM vitamins and 1 mmol/L sodium pyruvate (all from Life Technologies). Lipopolysaccharide (LPS) from *Escherichia coli* O127:B8 and O155:B5 (Sigma–Adrich) were used for cell cultures and animal models, respectively. Pam3CSK4 (InvivoGen) as TLR2 ligand and a combination of TNFα and IL-1β (both from PeproTech) as a maturing factors (MFs) were used. CBR agonists WIN55212-2 (Sigma Aldrich) and HU210, selective CB2 agonist HU308, selective antagonists for CB1 (Rimonabant), CB2 (AM630), PPARα (GW6471), PPARγ (GW9662) (all from Tocris) and autophagy inhibitor 3-methyladenine (3-MA; InvivoGen) were used.

### HmoDCs generation, naive CD4^+^ T cells purification and total blood DCs isolation

Peripheral blood mononuclear cells (PBMC) were obtained from buffy coats of healthy donors (source: Transfusion Centre of Madrid) by Ficoll-Paque Plus (GE-Healthcare) density gradient centrifugation. Immature hmoDCs were generated from blood monocytes obtained from total PBMC using anti-CD14 microbeads (Miltenyi Biotec) and cultured for 6 days with cRPMI medium containing 100 ng/mL of IL-4 and GM-CSF (PeproTech). The purity and phenotype of monocytes and generated immature hmoDCs were analysed by flow cytometry with lineage-specific markers. Purified naïve CD4^+^ T cells and total dendritic cell fraction were isolated from PBMC using the “Naïve CD4^+^ T Cell Isolation Kit” and “Blood Dendritic Cell Isolation Kit II”, respectively (Miltenyi Biotec). All isolations were performed in autoMACS Pro according to manufacturer´s protocol.

### Cell cultures

For titration experiments, immature hmoDCs from healthy donors (10^6^ cells/mL) were treated with LPS (0.1 μg/mL) plus different doses of WIN55212-2, HU210 or HU308 (1, 5, 10 and 20 μM) or with TLR2L (25 ng/mL) or MFs (25 ng/mL IL-1β and 50 ng/mL TNFα) plus different doses of WIN55212-2 (5, 10 and 20 μM).

Immature hmoDCs or human total blood DCs from healthy donors (10^6^ cells/mL) were stimulated with medium (unstimulated), WIN55212-2 (10 µM), LPS (0.1 μg/mL) or LPS plus WIN55212-2 for 18 h. Cells were used to analyse their phenotype by flow cytometry and cell‐free supernatants to quantify IL-8, IL‐1β, IL‐6, TNFα and IL‐10 by sandwich enzyme-linked immunosorbent assay (ELISA). For inhibition experiments, hmoDCs were preincubated for 1 h with Rimonabant (20 µM), AM630 (20 µM), GW6471 (25 µM), GW9662 (10 µM) or 3-MA (25 µM) or corresponding vehicle controls prior to activation. Then, cells were stimulated with LPS plus WIN55212-2 for 18 h in the presence of the corresponding inhibitors. Cell viability was analysed in all the cases by trypan blue (Gibco) exclusion and/or Annexin V and 7-AAD (Biolegend) by flow cytometry analysis.

### Coculture experiments

HmoDCs or human total blood DC fraction treated with medium (unstimulated), WIN55212-2 (10 μM), LPS (0.1 μg/mL) or the combination of LPS plus WIN55212-2 were cocultured with purified allogeneic naïve CD4^+^ T cells (DC:T cell ratio of 1:5) for 5 days. IL-17A, IFN-γ, IL-13, IL-5 and IL-10 were quantified in cell-free supernatants by ELISA.

### Tregs suppression assay

CD4^+^CD25^+^CD127^-^FOXP3^+^ Treg cells induced by allogeneic WIN55212-2 plus LPS-stimulated hmoDCs were purified by cell sorting of CD4^+^CD25^+^CD127^−^ population and mixed with CFSE-labelled autologous PBMC (responder cells) at different ratios and stimulated with plate-bound anti-human CD3 antibody (1 mg/mL, clone OKT3; eBioscience) and soluble anti-human CD28 (1 mg/mL, clone CD28.6; eBioscience) for 5 days. For control purposes, CFSE-labelled PBMCs were cultured alone with or without stimulation, and non-Treg cells (negative fraction of the sorting) were also tested. Proliferation of CD4^+^ T cells was determined by using CFSE dilution with flow cytometry.

### LPS-sepsis mouse model

All mice procedures included in this study were reviewed and ethically approved by Universidad Complutense de Madrid (UCM) and Comunidad Autónoma de Madrid (CAM) within the context of project SAF-2017-84978-R, (CAM:ref.10/250312.9/18). BALB/c mice (female, six-week-old, Charles River) were randomly divided into body weight-matched groups and intraperitoneally injected with vehicle (DMSO), WIN55212-2 (5 mg/kg), LPS (10 or 20 mg/kg), LPS plus WIN55212-2 or LPS with WIN55212-2 in the presence of Rimonabant (5 mg/kg) GW6471 (5 mg/kg) or 3-MA (15 mg/kg). The survival rate 78 h after LPS (20 mg/kg) administration was monitored. The weight loss was evaluated 24 h after LPS (20 mg/kg) administration. Serum samples and spleens were collected from mice at 16 h after LPS (20 mg/kg) administration. For mechanistic studies, assessment score were monitored and spleens were collected 72 h after LPS (10 mg/kg) administration. For Treg depletion, mice were intraperitoneally injected with 250 µg of anti-CD25 antibody (clone PC61, Biolegend) 8 days before LPS administration. Tregs depletion was confirmed by quantification of spleen Tregs 7 days after anti-CD25 administration by flow cytometry.

### Serum collection

Mice were anaesthetised with isoflurane, and peripheral blood was collected via retro-orbital bleeding using lime grass Pasteur pipettes and microtainer tubes (Beckton Dickinson). Samples were centrifuged at 10,000 rpm for 10 min at room temperature. Serum was collected and stored at −80 °C for further analysis. Concentrations of IL-6 were quantified by sandwich ELISA using specific ELISA cytokine kits for each one (BD Biosciences).

### Spleen processing and DCs isolation

Spleens were minced and filtered through 40 μm nylon strainers to obtain a single-cell suspension. Then, red blood cells were lysed with ACK lysis buffer before being resuspended in cRPMI and assessing splenocyte viability using Trypan Blue exclusion. Mice DCs from spleen were isolated using “Pan Dendritic Cell Isolation Kit, mouse” (Miltenyi). The purity and phenotype of DCs were analysed by flow cytometry with lineage-specific markers: MHCII, CD11c and CD86. Autophagy and cytokine induction in purified spleen DCs was evaluated by analysing mRNA levels of the genes IL-6, IL-10, ATG5, ATG12, ATG14 and LC3 by real time qPCR.

### Assesment score

The physical appearance of LPS-induced septic mice were scored according to the appearance of coat, activity and posture. The point of coat: 1, smooth; 2, mild ruffling; 3, significant ruffling. The point of activity: 1, normal; 2, moves slowly; 3, minimal movement with stimulation. The point of posture: 1, moving or resting normally; 2, resting together; 3, huddled. The assessment score is the total sum of the points from the above categories.

### Peanut-induced anaphylaxis mouse model

#### Epicutaneous sensitisation

A total of 100 µg of crude peanut extract (CPE, 10 mg/mL) (Greer Laboratories) was directly applied onto shaved and tape-stripped skin for 10 consecutive days.

#### Challenge

2.5 g of CPE alone or CPE plus 20 µg of WIN55212-2 in 500 µL of PBS was injected intraperitoneally 2 weeks after the last sensitisation treatment. Mice were monitored for 40 min after challenge to assess anaphylactic response:

1. Clinical score. Anaphylactic symptom score were obtained by evaluating the following symptoms: hind leg scratching in ear canal, reduced movement, puffy eyes, and convulsion. The presence of each symptom was pointed with 1 point and the total score was the sum of the different points.
2. Core body temperature. Rectal temperature readings were performed every 10 min with a rectal probe digital thermometer (VWR).
3. Hemoconcentration. Peripheral blood were collected 40 min after challenge by retro-orbital bleeding into microcapillaries. The tubes were centrifuged for 5 min by using a microhematocrit centrifuge (QuercusLab). The hematocrit value was expressed as a percentage of cell volume.

### Serum peanut-specific immunoglobulins

Peanut-specific IgE and IgG_1_ were measured by ELISA. High binding 96-well plates (Corning) were coated with CPE (20 μg/mL) in coating buffer at 4 °C overnight. Coated plates were blocked with FBS (10%) in PBS for 2 h at room temperature. Plates were washed and incubated with serum samples overnight at 4 °C. After washing, detection antibody (rat anti-mouse IgE-HRP (Southern Biotech) or goat anti-mouse IgG_1_-HRP (Thermo Fisher Scientific)) was added and incubated for 2 h at room temperature. *O*-phenylenediamine (OPD) was used to develop the assay and H_2_SO_4_ (3 N) was added to stop the reaction for absorbance reading at 492 nm.

### Splenocyte cell culture

Spleens were processed and triplicates of 0.8 × 10^6^ splenocytes were cultured in medium alone or with CPE (250 µg/mL) in flat-bottom 96-well plates (Corning). After 5 days of culture at 37 °C and 5% CO_2_, the triplicates were pooled, cells were collected for FOXP3 staining, and cell-free supernatants were used to quantify IFNγ, IL‐5 and IL‐10 by ELISA.

### Statistical analysis

Statistical analyses were performed using GraphPad Prism software, version 6.0. All the data were expressed as mean ± SEM of the corresponding parameter. Statistical analysis was calculated using One-way ANOVA or Paired *t* test. Differences were considered statistically significant when *P* < 0.05.

## Supplementary information

Online Supplementary Methods
