# Supplementary material for: Cannabinoids induce functional Tregs by promoting tolerogenic DCs via autophagy and metabolic reprograming
Source: Mucosal Immunol. 2021 Sep 21;15(1):96–108. doi: 10.1038/s41385-021-00455-x (PMC8732281; doi:10.1038/s41385-021-00455-x)
Supplement: Supplementary file 1 — Online Supplementary Methods [file 41385_2021_455_MOESM1_ESM.pdf]

TITLE PAGE

Cannabinoids for inflammatory diseases: functional Tregs induction by promoting tolerogenic DCs via autophagy and metabolic reprograming

Alba Angelina[1], Mario Pérez-Diego[1], Jacobo López-Abente[1], Beate Rückert[2], Ivan Nombela[1], Mübeccel Akdis[2], Mar Martín-Fontecha[3], Cezmi Akdis[2], Oscar Palomares[1]

[1] Department of Biochemistry and Molecular Biology, School of Chemistry, Complutense University of Madrid, Madrid, Spain.
[2] Swiss Institute of Allergy and Asthma Research (SIAF), University of Zürich, Davos, Switzerland.
[3]Department of Organic Chemistry, School of Chemistry, Complutense University of Madrid, Madrid, Spain.

Corresponding Author: Oscar Palomares, PhD
Department of Bichemistry and Molecular Biology,
School of Chemistry, Complutense University of Madrid,
Avenida Complutense s/n,
28040 Madrid, Spain
Telephone: +34 913944159
Email: oscar.palomares@quim.ucm.es

Online Supplementary Methods

Flow cytometry and confocal microscopy

Cells were washed with PBS/EDTA (2 mM) 0.5% BSA and labelling of cell-surface markers was performed at room temperature for 15 minutes in darkness with fluorescence-labelled antibodies (**Table S1**) or corresponding isotype controls. CB1 staining was performed following previously described protocols(*1*). Briefly, cells were incubated with the previously validated Alexa488-HU210 fluorescence probe or Alexa488-alkyne as control at a final concentration of 1µM in PBS for 30 minutes at room temperature with shaking and darkness. For CB2 staining, cells were incubated with anti-CB2 antibody or its corresponding isotype control for 30 minutes at room temperature. After wash with PBS, cells were incubated with the secondary antibody (anti-rabbit labelled with Alexa488) for 30 minutes at room temperature and darkness. Type 2 cDCs and pDCs contained in the total DC fraction were characterized by the high expression of HLA-DR and the lack of CD19 expression. Within the HLA-DR^{high} total DCs fraction, type 2 cDCs were identified as CD1c^{+}, whereas pDCs as CD303^{+} and CD123^{+}. For analysis of FOXP3 expression in human T cells primed by DCs, cells were first subjected to surface staining. After fixation and permeabilization, cells were stained with anti-human FOXP3-Alexa Fluor 488, according to manufacturer's recommendations. The same protocol described above was carried out for the phenotypic characterization of CD4^{+}CD25^{high}FOXP3^{+} Treg cells in freshly isolated mouse splenocytes. For, IL-10 and FOXP3 analysis, the primed CD4^{+} cells were washed and re-stimulated with 25 ng/mL PMA plus 1 µg/mL ionomycin for 6 hours and Brefeldin A (10 µg/mL) for last 4 hours. For autophagy analysis, autophagosomes were detected using Autophagy detection kit (Enzo Life Sciences) according to the manufacturer´s instructions. Briefly, cells were incubated with CYTO-ID green autophagy detection dye for 30 minutes at 37 ºC. Subsequently, cells were washed and analysed. Flow cytometry analysis was performed in a FACScalibur cytometer or FACS Aria III (both from Beckton Dickinson) in the

Cytometry and Fluorescence Microscopy Unit at Complutense University of Madrid. For confocal microscopy, stained cells were subjected to cytospin, fixed with ice-cold methanol for 5 minutes, and stained with ProLong Gold–DAPI (Dako) 24 hours before analysis. Images were acquired and analyzed by using the confocal microscope LMS 780 Zeiss (Carl Zeiss Microscopy GmbH).

Cytokine quantification

Concentrations of TNFα, IL-8, IL-6, IL-1β, IL-10, IFN-γ, IL-13 and IL-5 in cell-free supernatants were quantified by sandwich ELISA using specific ELISA cytokine kits for each one (BD Biosciences). IL-17A levels were quantified by the IL-17 quantikine Elisa Kit (RD Systems). In all cases, manufacturer's instructions were followed with minor modifications.

Metabolic studies

The Warburg effect in stimulated hmoDC cultures was determined photometrically 18 hours after stimulation by quantifying the optical density (OD) at 570 nm and calculating the Warburg effect as $1/OD_{570nm}$ normalized to the unstimulated condition. Lactate concentrations in culture supernatants were determined 18 hours after stimulation by using the colorimetric L-Lactate Assay kit (Abcam), according to the manufacturer's recommendations. Glucose concentrations in culture supernatants were determined 18 hours after stimulation by using the Glucose (GO) Assay Kit (Sigma-Aldrich). The metabolic rate was derived mathematically in percentage of medium without DCs (glucose concentration in cRPMI 1640 = 2 mg/mL). To measure mitochondrial membrane potential, the fluorescent dye MitoTracker Red CMXRos was used (250 nM, Thermo Fisher Scientific). ATP concentrations in lysates of DCs were determined by using ATP Determination Kit (Invitrogen). For real-time metabolic characterization, the mitochondrial oxygen consumption rate (OCR, pmol/min) was analysed using a Seahorse XF HS Mini Analyzer (Agilent). For OCR studies, hmoDCs were harvested, washed and resuspended in DMEM medium supplemented with 10 mM glucose, 1 mM

pyruvate and 2 mM glutamine. Cells (50 x 10^3/well) were plated in poly-D-lysine-coated 8-well plates and incubated in a non-CO_2 incubator for 1 hour at 37ºC. A complete OCR study was performed in four consecutive stages: basal respiration (without drugs), mitochondrial complex V inhibition (1 µM oligomycin), maximal respiration induction (1 µM carbonyl cyanide 4-(trifluoromethoxy)phenylhydrazone (FCCP)), and electron transportation chain inhibition (0.5 µM rotenone and 0.5 µM antimycin A).

Western blotting

Freshly isolated hmoDCs were treated with medium (unstimulated), WIN55212-2 (10 µM), LPS (0.1 µg/mL) or the combination of WIN55212-2 and LPS. For inhibition experiments, cells were preincubated during 1 hour with Chlorochine (20 µM), E64d plus Pepstatin A (both at 10 µg/mL), Rimonabant (20 µM) or GW6471 (25 µM). After 15, 30 minutes or 18 hours at 37°C, cells were harvested and lysed with RIPA buffer (Thermo Fisher scientific) supplemented with Protease/Phosphatase Inhibitor Cocktail (Cell Signaling) for 30 minutes at 4°C. Lysates were centrifuged for 15 minutes at 10,000 g and 4°C, and supernatants were collected for protein quantification with Micro BCA Kit (Pierce, Rockford, Ill). Ten micrograms of total protein from cell lysates was separated by means of SDS-PAGE and transferred onto a nitrocellulose or PVDF membrane (Bio-Rad Laboratories). The membrane was blocked with 5 % BSA, 0.1 % Tween-20 in TBS for 1 hour and incubated with primary antibodies (**Table S2**). Then, the membrane was washed and incubated with goat anti-rabbit (1/4000; Bio-Rad Laboratories) or goat anti-mouse (1:2500, Pierce) conjugated with horseradish peroxidase as a secondary antibody. Reactive bands were visualized with the ECL chemiluminescence system (Bio-Rad Laboratories) in a Fujifilm LAS-3000 developer. The OD of the reactive bands was quantified with Fujifilm Multi Gauge software, and values were expressed relative to the β-actin loading control.

RNA extraction, cDNA synthesis, and quantitative real-time PCR

RNA was isolated from harvested cells stimulated with the different stimulus for 4 hours using the RNeasy mini kit (Qiagen) according to the manufacturer's instructions. cDNA was generated using a PrimeScript RT reagent Kit (Takara). Real-time quantitative PCR was performed on cDNA using FastStart Universal SYBR Green Master (Rox) (Roche). The sequences of the employed pair primers are shown in **Table S3**. Samples were run on a real-time PCR system (ABI Prism 7900 HT; Applied Biosystems). Data were expressed as arbitrary units (A.U.), which are $2^{-(\Delta CT)}$ values multiplied by 10^4, where ΔCT correspond to the difference between the cycle threshold value for the gene of interest and the housekeeping gene, EF1α or GAPDH.

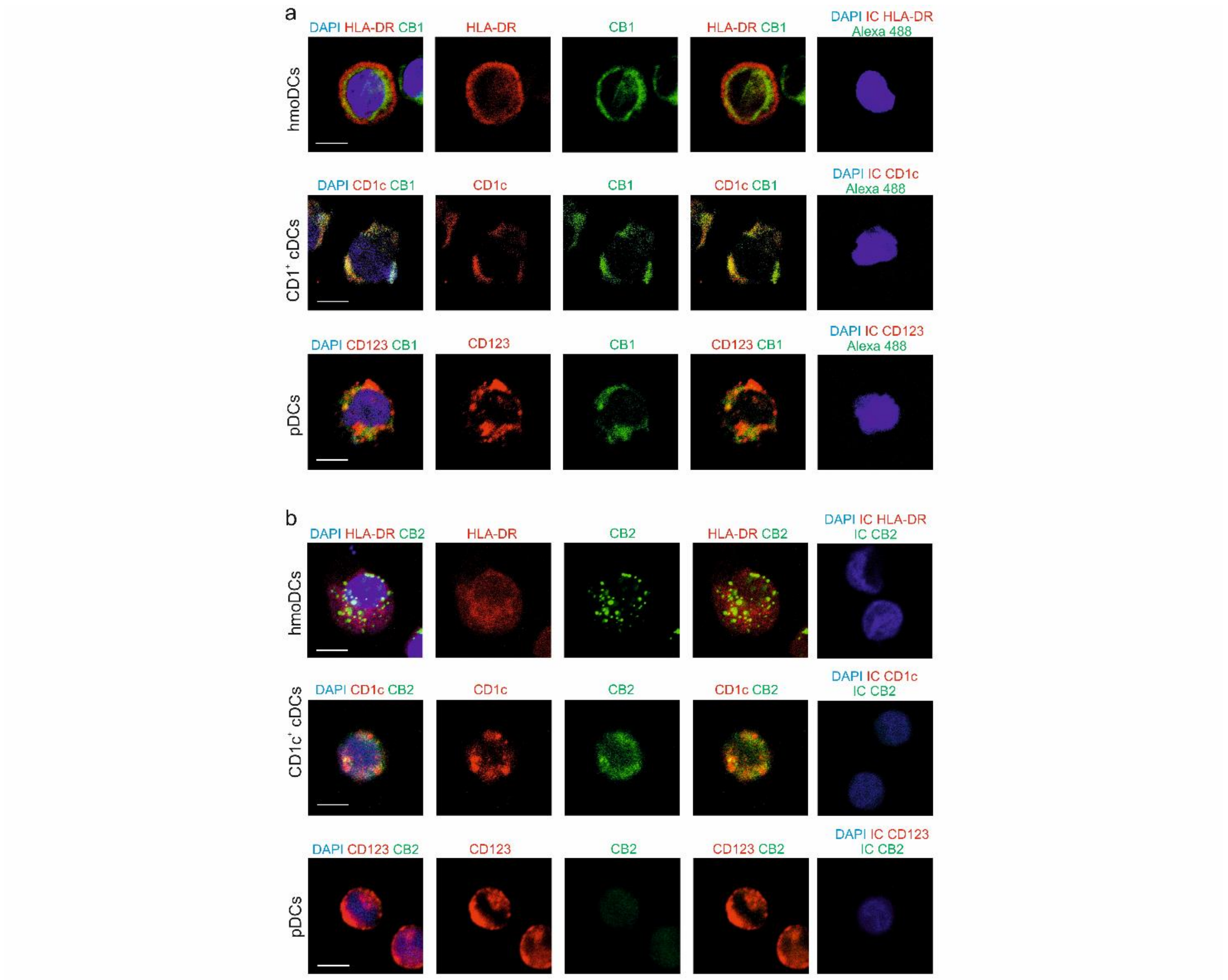

Supplementary Fig. 1. Human DCs express CB1 and CB2 at the protein level. **a** and **b** Representative confocal images of CB1 and CB2 expression in hmoDCs and human blood CD1c+ cDCs and pDCs. DAPI (*blue*), HLA-DR, CD1c or CD123 (*red*) and CB1 or CB2 (*green*). White bars, 5 µm. IC, isotype control.

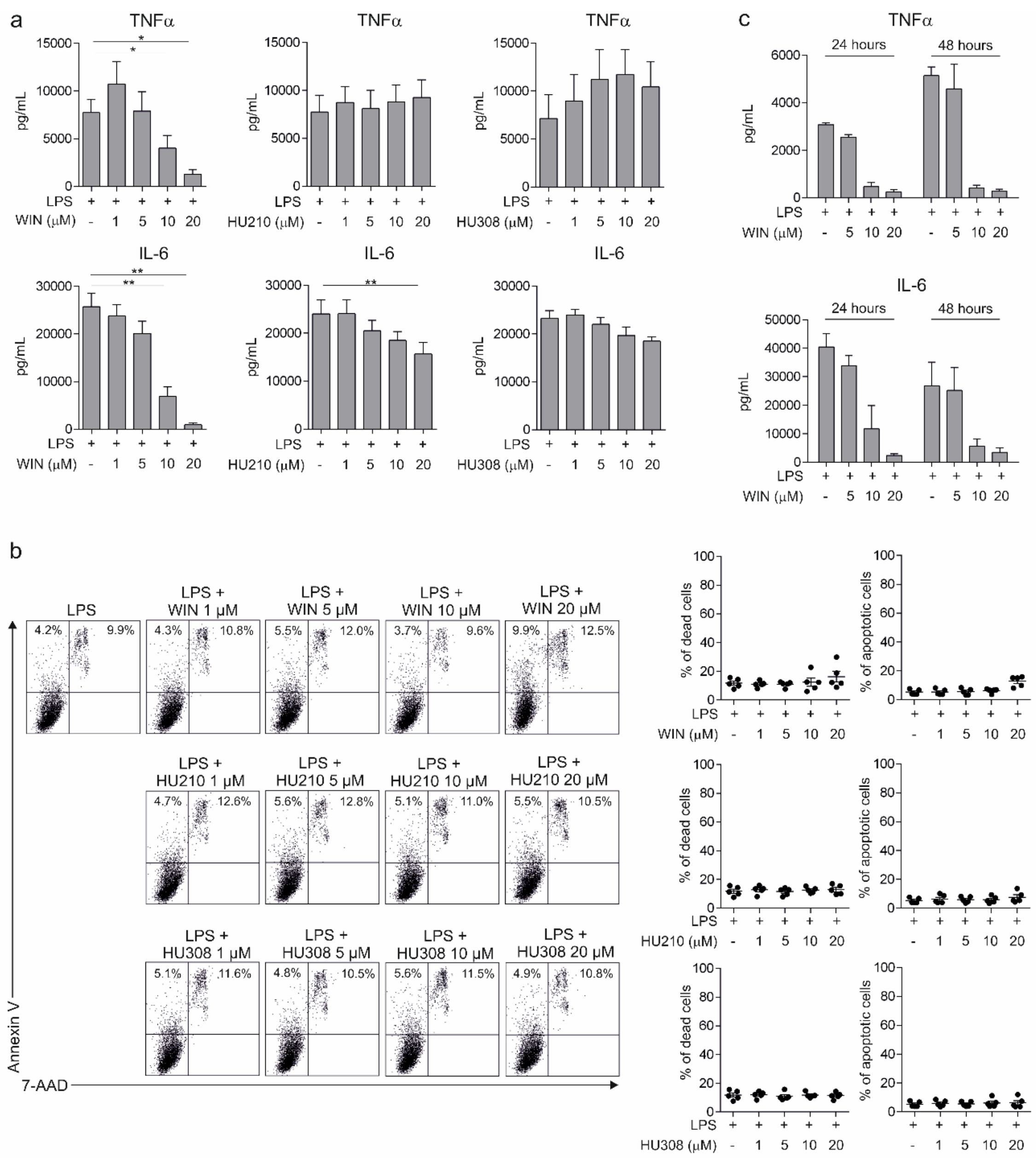

Supplementary Fig. 2. Synthetic cannabinoids modulate hmoDCs function. **a** Cytokine production after stimulation of hmoDCs with LPS (0.1µg/mL) or LPS plus different doses of WIN55215-2 (WIN), HU210 or HU308 (n = 3-4)**. b** Representative flow cytometry dot plots for 7-AAD and Annexin V and percentage of dead cells and apoptotic cells after 18 hours of stimulation with the indicated conditions is shown (n = 5). **c** Cytokine production after stimulation of hmoDCs with LPS plus different doses of WIN55212-2 at 24 or 48 hours (n = 3). Values are the mean ± SEM. Statistical significance was determined using One-way Anova. * *P* < 0.05.

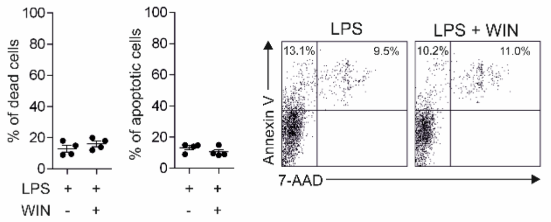

Supplementary Fig. 3. The synthetic cannabinoid WIN55212-2 does not induce cell death in total DCs from peripheral blood. Representative flow cytometry dot plots for 7-AAD and Annexin V are shown.

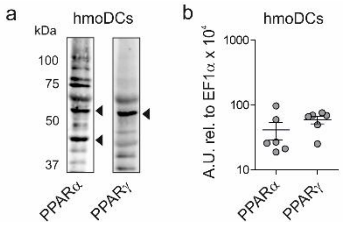

Supplementary Fig. 4. hmoDCs express PPARα and PPARγ at the protein and mRNA level. **a** Western blot of PPARα and PPARγ expression in hmoDCs. One representative example of three independent experiments. **b** mRNA expression levels of PPARα and PPARγ in hmoDCs (n = 6).

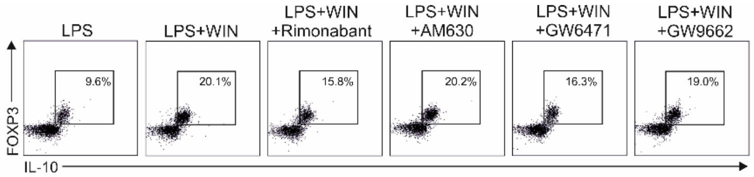

Supplementaty Fig.5. Tolerogenic DCs generated by cannabinoids induce IL-10 producing FOXP3^{+} Tregs. Representative flow cytometry dot plos for IL-10 and FOXP3 are shown.

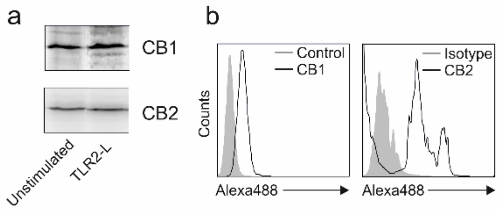

Supplementary Fig. 6. THP1-XBlue™ cell line express CB1 and CB2. **a** Western blot of CB1 and CB2 expression in THP1-XBlue™ cells. **b** Flow cytometry histograms of CB1 and CB2 expression in THP1-XBlue™ cells. Grey shadowed lines represent the controls and black empty lines represent CB1 or CB2 staining.

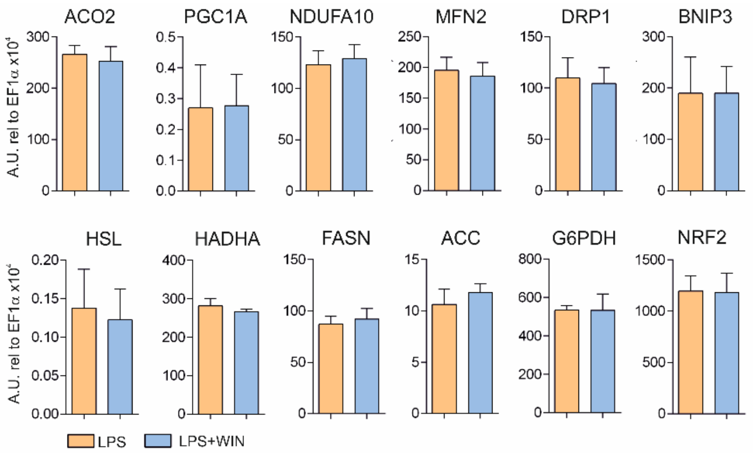

Supplementary Fig. 7. mRNA expression levels of different metabolism-related genes. ACO2, aconitate hydratase 2; PGC1A, peroxisome proliferator-activated receptor gamma coactivator 1 alpha; NDUFA10, NADH dehydrogenase 1 alpha subcomplex subunit 10; MFN2, mitofusin 2; DRP1, dynamin-1-like protein; BNIP3, BCL2 interacting protein 3; HSL, hormone-sensitive lipase; HADHA, hydroxyacyl-CoA dehydrogenase trifunctional multienzyme complex subunit alpha; FASN, fatty acid synthase; ACC, acetyl-CoA carboxylase; G6PDH, glucose-6-phosphate 1 dehydrogenase; NRF2; nuclear factor erythroid 2-related factor.

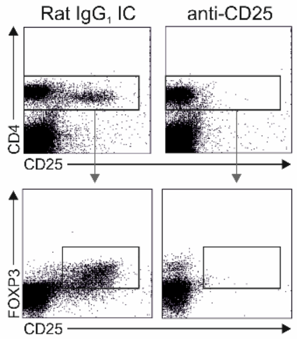

Supplementary Fig. 8. Treg depletion in BALB/c mice using anti-CD25 antibody. IC, Isotype control.

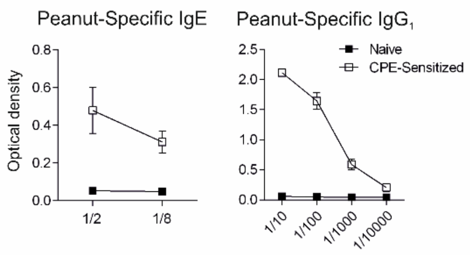

Supplementary Fig. 9. Epicutaneously CPE-sensitized mice show high levels of peanut-specific IgE and IgG1 as determined by ELISA. CPE, crude peanut extract.

Supplementary Table 1: Flow cytometry antibodies

Name	Supplier	Clone	Conjugate
**Human FcR Blocking**	Miltenyi	-	-
**Human HLA-DR**	Biolegend	L243	FITC, APC
**Human CD86**	Miltenyi	FM95	PE
**Human CD83**	Miltenyi	HB15	APC
**Human CD127**	Biolegend	A019D5	PE
**Human FOXP3**	Biolegend	259D	Alexa488
**Human CD4**	Biolegend	OKT4	PerCP
**Human CD25**	Biolegend	BC96	APC
**Human CB2**	Invitrogen	-	-
**Human CD123**	Biolegend	6H6	FITC
**Human CD1c**	Biolegend	L161	FITC
**Human CD3**	BD Pharmingen	OKT3	APC
**Human CD19**	Biolegend	HIB19	PE/Cy7
**Human CD303**	Miltenyi	AC144	PE
**Human IL-10**	Biolegend	JES3-19F1	PE
**Mouse FOXP3**	Biolegend	MF-14	Alexa488
**Mouse CD25**	Biolegend	PC61	PE
**Mouse CD4**	Biolegend	GK1.5	PerCP
**Mouse CD11c**	Biolegend	N418	APC
**Mouse MHCII**	Biolegend	M5/114.15.2	PE/Cy7
**Mouse CD86**	Biolegend	GL-1	BV605

Suplementary Table 2: Western blot antibodies

Name	Dilution	Supplier
PPARα	1:1000	Invitrogen
PPARγ	1:1000	Cell Signaling
Phosho-IKKα/β (Ser176/177)	1:1000	Cell Signaling
Phospho-IκBα (Ser32/35)	1:1000	Cell Signaling
Phospho-p38 MAPK (Thr180/182)	1:1000	Cell Signaling
Phospho-ERK (Thr202/Tyr204)	1:1000	Cell Signaling
Phospho-SAPK/JNK (Thr183/Tyr185)	1:1000	Cell Signaling
Phospho-p70S6K(Thr389)	1:500	Cell Signaling
Phospho-Akt (Ser473)	1:1000	Cell Signaling
Phospho-AMPK (Thr172)	1:1000	Cell Signaling
LC3-I/II	1:2000	Sigma-Aldrich
B-Actin	1:15000	Sigma-Aldrich
**Name**	Dilution	Supplier
PPARα	1:1000	Invitrogen
PPARγ	1:1000	Cell Signaling
Phosho-IKKα/β (Ser176/177)	1:1000	Cell Signaling
Phospho-IκBα (Ser32/35)	1:1000	Cell Signaling
Phospho-p38 MAPK (Thr180/182)	1:1000	Cell Signaling
Phospho-ERK (Thr202/Tyr204)	1:1000	Cell Signaling
Phospho-SAPK/JNK (Thr183/Tyr185)	1:1000	Cell Signaling
Phospho-p70S6K(Thr389)	1:500	Cell Signaling
Phospho-Akt (Ser473)	1:1000	Cell Signaling
Phospho-AMPK (Thr172)	1:1000	Cell Signaling
LC3-I/II	1:2000	Sigma-Aldrich
B-Actin	1:15000	Sigma-Aldrich

Supplementary Table 3: primers for real-time qPCR

Name	Forward	Reverse
EF1α	CTGAACCATCCAGGCCAAAT	GCCGTGTGCAATCCAAT
**IL-8**	GCAGCTCTGTGTGAAGGTGCAGTT	TTCTGTGTTGGCGCAGTGTGGTC
IL-1β	TTTTTGCTGTGAGTCCCGGAG	TTCGACACATGGGATAACGAGG
IL-6	GGTACATCCTCGACGGCATCT	GTGCCTCTTTGCTGCTTTCAC
IL-10	GTGATGCCCCAAGCTGAGA-	CACGGCCTTGCTCTTGTTTT
PPARα	CATTACGGAGTCCACGCGT	ACCAGCTTGAGTCGAATCGTT
PPARγ	GAACAGATCCAGTGGTTGCAG	CAGGCTCCACTTTGATTGCAC
ATG5	GCAGATGGACAGTTGCACACAC	GAGGTGTTTCCAACATTGGCTCA
ATG12	TAGAGCGAACACGAACCATCC	CACTGCCAAAACACTCATAGAGA
ATG14	GAGGAAGTAAAGACGGGTGTG	CCATTGTTAGGGAGGCTAATCC
PIK3C3	GGGATTAGTGCTGAGGTCATG	AGTCTATGTGGAAGAGTTTGCC
ULK1	GCAAGGACTCTTCCTGTGACAC	CCACTGCACATCAGGCTGTCTG
RUBCN	AGGCCCCAGGAATATCACCA	TCTTCGGAACATGCCTTCCC
BECN1	CCATGCAGGTGAGCTTCGT	GAATCTGCGAGAGACACCATC
ATG16L	TGCAGTTCAGTCCAGGTTC	GCTAAGAGGTAAGATCCAGCAC
GLUT1	GGCTTCTCCAACTGGACCTC	CCGGAAGCGATCTCATCGAA
HK2	TTCGCACTGAGTTTGACCAG	TCACCAGGATAAGCCTCACC
PFKFB3	AGCCCGGATTACAAAGACTGC	GGTAGCTGGCTTCATAGCAAC
LDHA	ATGGCAACTCTAAAGGATCAGC	CCAACCCCAACAACTGTAATCT
HIF1A	TCGCATCTTGATAAGGCCTCT	ACAAAACCATCCAAGGCTTTCA
PDHA1	ATGGAATGGGAACGTCTGTTG	CCTCTCGGACGCACAGGATA
IDH3A	AGGTTTTACTGGTGGTGTTCA	CCTCCCACTGAATAGGTGCTT
SDHA	CGAACGTCTTCAGGTGCTTT	AAGAACATCGGAACTGCGAC
ATP5A1	AAGACACGCCCAGTTTCTTC	TTTGGGTTCATCTTTCATTGC
PINK1	CCCAAGCAACTAGCCCCTC	CCCAAGCAACTAGCCCCTC
ACADM	GGAAAGCTACTTGTAGAGCACCA	ACGACCAGAATCAACCTCCC
CPT1A	GACTCTGGAAACGGCCAACT	ATCTTGCCGTGCTCAGTGAA
GLS	GCAGTCTGGAGGAAAGGTTGC	CACACCCCACAAATCGGGAC
SLC1A3	CTGGGGACCTCTTCAAGTTCTG	GGGAGCACGAATCTGGTGAC
ACO2	AATGGATGTACTCGTTGGGC	ACAGCCTACTGGTGACTCGG
PGC1A	CCTGACACAACACGGACAGA	AACCATAGCCATCATCCCGC
NDUFA10	ACAGAACGCAGCAGAGTGATA	GGAAAGTGCTTGAAGCCTAGTT
MFN2	TCTGGGACCTTTGCTCATCT	CAACCAACCGGCTTTATTCC
DRP1	CTATCTGCTCGGGAACAGCG	TACTGCCTTTGGCACACTGTC
BNIP3	TCAGCATGAGGAACACGAGC	ACGCCTTCCAATATAGATCCCCAA
HSL	CTCAGTGTGCTCTCCAAGTG	CACCCAGGCGGAAGTCTC
HADHA	CTCTCCCGTGGACAAGATGC	AAGCCAGGTCCATCCTTAACC
FASN	CTGCACTTCCATAGCCCCAA	AAGGAGTTGATGCCCACGTT
ACC	ACAACGCAGGCATCAGAAGA	GTTTCACCGCACACTGTTCC
G6PDH	AAGCCCATCCCCTATATTTATGGC	GGTGCCCTCATACTGGAAACC
NRF2	AGGTTGCCCACATTCCCAAA	AACGTAGCCGAAGAAACCTCA
mIL-6	GAAACCGCTATGAAGTTCCTCTCTG	GTATCCTCTGTGAAGTCTCCTCTCC
mIL-10	GGCCCAGAAATCAAGGAGCA	AGGGGAGAAATCGATGACAGC
mATG5	GCAGATGGACAGTTGCACACAC	GAGGTGTTTCCAACATTGGCTCA
mATG12	TAGAGCGAACACGAACCATCC	CACTGCCAAAACACTCATAGAGA
mATG14	AACCCAAGGACCTGACATGGA	ACACAATGTTGACGAGCTGC
mLC3A	CGCTACAAGGGTGAGAAGCA	GCGGCGCCGGATGAT
mGAPDH	TCCTGCACCACCAACTGCTTA	GCCATCACGCCACAGCTTTC

1. M. Martin-Fontecha *et al.*, A Fluorescent Probe to Unravel Functional Features of Cannabinoid Receptor CB1 in Human Blood and Tonsil Immune System Cells. *Bioconjug Chem* **29**, 382-389 (2018).
